# Ionotropic Gelation of Chitosan Flat Structures and Potential Applications

**DOI:** 10.3390/molecules26030660

**Published:** 2021-01-27

**Authors:** Pasquale Sacco, Seidy Pedroso-Santana, Yogesh Kumar, Nicolas Joly, Patrick Martin, Patrizia Bocchetta

**Affiliations:** 1 Department of Life Sciences, University of Trieste, Via Licio Giorgieri 5, I-34127 Trieste, Italy; psacco@units.it; 2 Pathophysiology Department, School of Biological Sciences, Universidad de Concepción, 4030000 Concepción, Chile; spedroso@udec.cl; 3 Department of Physics, ARSD College, University of Delhi, Delhi 110021, India; ykumar@arsd.du.ac.in; 4 Unité Transformations & Agroressources, Université d’Artois—UniLasalle, ULR7519, F-62408 Béthune, France; nicolas.joly@univ-artois.fr (N.J.); patrick.martin@univ-artois.fr (P.M.); 5 Dipartimento di Ingegneria dell’Innovazione, Università del Salento, Via Monteroni, 73100 Lecce, Italy

**Keywords:** carbohydrate polymers, chitosan, ionotropic gelation, chitosan membranes, flat chitosan

## Abstract

The capability of some polymers, such as chitosan, to form low cost gels under mild conditions is of great application interest. Ionotropic gelation of chitosan has been used predominantly for the preparation of gel beads for biomedical application. Only in the last few years has the use of this method been extended to the fabrication of chitosan-based flat structures. Herein, after an initial analysis of the major applications of chitosan flat membranes and films and their usual methods of synthesis, the process of ionotropic gelation of chitosan and some recently proposed novel procedures for the synthesis of flat structures are presented.

## 1. Introduction

Chitosan (Chit) flat structures have not been yet classified in literature and thus, reviews on their methods of synthesis, properties and applications are still lacking. Chit flat structures are a subset of Chit membranes and films and their applications range from wastewater treatments, food packaging and biomedical uses to novel energy conversion and storage devices. Every method of synthesis possesses advantages and disadvantages depending on the physico-chemical, structural and shape/size features of the chitosan-based flat structures required for a specific application.

Since the 1990s, ionotropic gelation has been used in the synthesis of polymeric micro and nanoparticles for biomedical applications [1]. Chitosan structures encapsulating doxorubicin [2], interferons [3,4], antioxidants [5] and other molecules [6] have been obtained, demonstrating the versatility of this technique. A relatively simple procedure, its flexibility to produce particles in a wide range of sizes, medium to high drug encapsulation efficiency, stable particles in suspension and the use of biocompatible and biodegradable polymers are among the reasons favoring the use of ionotropic gelation methods in biomedicine. The method has been intensively studied for gel beads and particles, allowing the development of many industries devoted to their production [7]. However, the capability of ionotropic gelation technology to entrap a great number of functional materials in chitosan has not been sufficiently explored by the scientific literature in other fields of application, such as energy conversion and storage, where flat structures are needed.

This review aims to shed light to the ionotropic gelation method mechanism for the synthesis of chitosan flat structures. After a brief introduction to the properties of chitosan, the methods of synthesis and application of chitosan flat structures are reviewed with a detailed discussion of recent research studies. Novel procedures introduced in the last years based on chitosan ionotropic gelation methods to synthesize chitosan flat structures for both biomedical [8] and energy conversion applications [9] are commented.

## 2. An Overview of Chitosan Properties and Methods of Synthesis of Flat Structures

### 2.1. Chitosan

**Figure 1:**
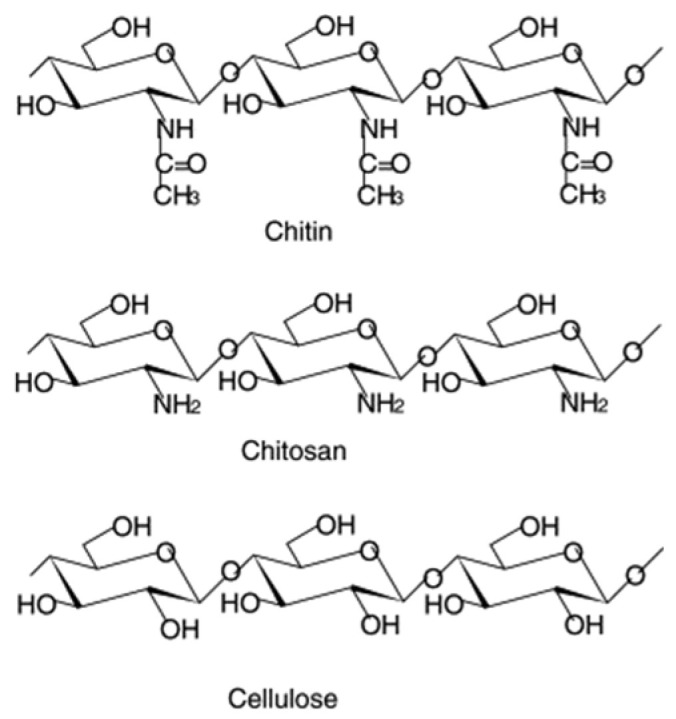
Structures of chitin, chitosan, and cellulose. Reprinted from [33] with permission from Elsevier.

Chitosan is considered the most versatile biopolymer among the polymer classes amenable of ionic crosslinking [10]. This preference is related to the inherent characteristics of this biopolymer: (i) abundance due to its derivation from chitin, the most copious polysaccharide in Nature, by alkaline deacetylation; (ii) renewability, because chitin is extracted from insects, molluscs and fungal mycelia [11], (iii) low-cost and environmentally friendliness. In addition, the chemical structure of Chit permits: (i) good water solubility at slightly low pH thanks to the presence of amine groups [8,12,13]; (ii) great film-forming ability thanks to the easy formation of intra- and inter-molecular hydrogen bonds [14] and (iii) facile chemical modification ascribed to the presence of diverse functional groups (-OH, -NH_2_, C-O-C) (Figure 1). From a structural point of view, Chit is well known to behave as a semi-flexible rod (or stiff coil), with a calculated persistence length, *l_p_*, of ~ 16 nm down to 7 nm, as previously demonstrated [15]. These properties allow the development of easy procedures for gel formation [16] and chemical modification of Chit-based structures [17,18,19].

The increasing use of Chit combines its excellent properties with those of other materials to obtain composites that exhibit superior performance for specific applications. The incorporation of Chit to polymeric blends, multilayer coatings, scaffolds and nanostructures, is being actively studied nowadays [20]. In this way, the thermal and mechanical characteristics of chitosan have been improved by combination with clays, polylactic acid, cellulose nanofibers, and metal oxides, for food packaging applications [21]. Chitosan films and coatings can replace petroleum-based materials and offer biodegradability and positive bioregulatory features.

Uses in the biomedical area include Chit nanocomposites with metal and graphene oxides for hyperthermia therapy [22], combination with hydroxyapatite and carbon nanotubes to obtain bone implants with higher osteogenic and mechanical properties [23], and the synthesis of new Chit-based nanosystems for the slow release and protection of bioactive ingredients [24]. The deposition of highly resistant elements in a chitosan matrix for metal corrosion inhibition [25] and the absorption of heavy metals and dye remotion from water and industrial wastes [26,27], are other current applications of Chit-based composites. Also, composite materials made of chitosan and conducting polymers (CPs), such as polypyrrole [28] and polyaniline [29], have been intensively studied for applications where the aforementioned advantages of Chit and the electron conductivity of CPs can be exploited. These applications include electrochemically controlled drug delivery systems [30], flexible electronics and polymer electrolytes for battery and supercapacitor technology. In the first case, Chit and CP can confer on the composite the capability to switch on/off and/or manage the drug-releasing rate [31], in the last application Chit/CP composites benefit from improved mechanical strength, dielectric properties or ionic conductivity [32].

The cationic behavior of chitosan in acidic solutions and the induction of gelation reaction mediate interactions with other materials and the formation of composites. The importance of chitosan and chitosan-based structures in many fields of daily life is largely due to their multifunctionality and the advantages related to the properties of the composites obtained, which will be evidenced in subsequent sections.

### 2.2. Flat Chitosan Structures: Methods of Synthesis

**Figure 2:**
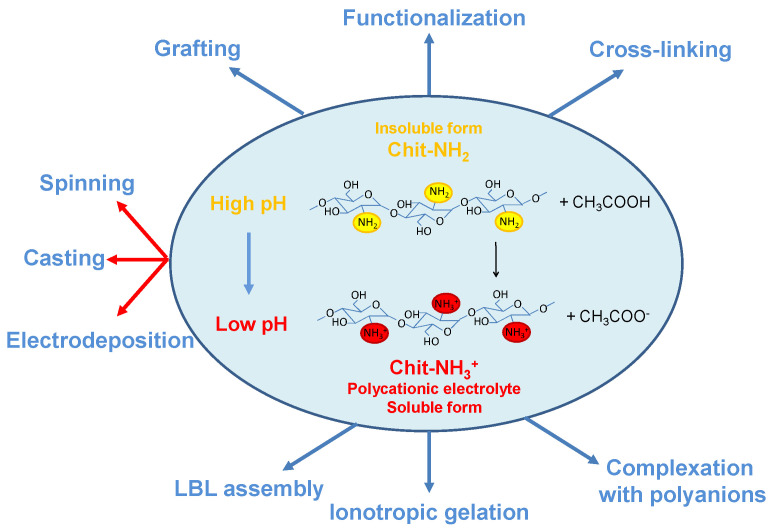
Scheme illustrating the chemical equilibrium of chitosan in slightly acidic solution and related methods to obtain chitosan flat structures from its soluble or insoluble form.

The capability of chitosan to turn from soluble to insoluble forms by changing the pH values is fundamental for the fabrication of composite chitosan structures with functionalities tailored for specific applications. The chemical reaction associated to this transformation is illustrated in Figure 2, together with the most important methods used to fabricate flat structures starting from soluble or insoluble forms. At a slightly low pH (for example in acetic acid) chitosan exists in the soluble form CS-NH_3_^+^ because of the protonation of its amine groups and breaking of hydrogen bonds. At neutral pH (ranging from 6.5 and 7.3 [34]), biocompatible values and thus attractive for biological applications, Chit becomes insoluble, forming Chit-NH_2_ precipitates because of the reduction of electrostatic repulsions and the formation of three-dimensional polymeric structures, such as films, fibers or hydrogels. As shown in Figure 2, the preparation of composite chitosan flat polymers can start from pre-formed insoluble chitosan as well as from chitosan polycation solutions. Depending of this initial condition several methods aimed at obtaining chemical, mechanical, or physical modifications of chitosan into specific shapes have been developed to synthesize and/or functionalize Chit-based polymer reticulates [35].

Chitosan flat structures (membranes or films) can be synthesized by many methods, the most representative of which are solvent evaporation, crosslinking, layer-by-layer assembly, electrodeposition, and ionotropic gelation. These techniques can be combined, and different follow-up treatments are also performed.

The most used technique for the synthesis of flat chitosan films is the solvent evaporation or solution-cast method [36,37,38,39] because its simplicity (Figure 2). The technique is based on the drying of a slightly acidic chitosan solution spread on a rigid support (Petri dishes, glass or plastic plates) at low or medium temperatures. Typical post-treatments of the obtained chitosan flat structures are: (i) neutralization with alkaline solutions and (ii) cross-linking. The latter is often done with glutaraldehyde [40]) to increase the mechanical strength of the polymer or with other crosslinkers capable of providing functionalities for specific applications [41,42]. This method was used in 2007 [41] to synthesize the first proton conducting chitosan-based membrane for application direct methanol fuel cells (DMFCs). After that, many other studies on this topic have used the solution-cast method to improve the proton conductivity of chitosan (see Section 3.2) and test the flat membranes in fuel cell apparatus. From an industrial point of view, this procedure is considered uneconomical and time-consuming, especially in the food packaging field [43].

**Figure 3:**
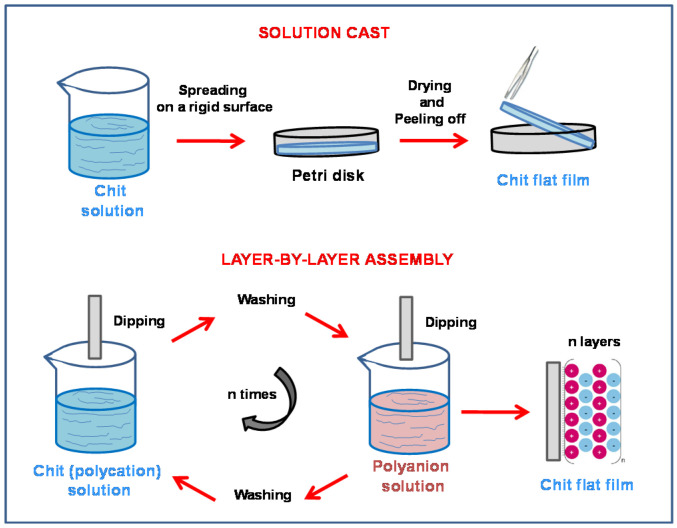
Scheme of solution-cast and LBL assembly methods for the fabrication of chitosan-based flat structures starting from a chitosan polycation solution (low pH, Chit-soluble form).

Chitosan flat structures deposited through layer-by-layer (LBL) self-assembly have been also studied for biomedical and energy conversion device applications [44]. LBL self-assembly is an easy technique employed to synthesize thin films by sequentially dip coating a substrate into negatively and positively charged polyelectrolyte solutions to exploit the electrostatic interactions between the two reagents (Figure 3) [45]. As discussed above, Chit is a cationic polyelectrolyte and thus can be coupled to anionic electrolytes to obtain LBL functional layers. The choice of relevant anionic compounds affects the final properties of the flat chitosan-based structures and their applications. Recently, LBL technology has been successfully used to produce proton conducting electrolytes for fuel cells [46,47]. However, in most of the cases the substrate used is made of expensive Nafion^®^ and, thus novel studies exploring the possibility to use low-cost chitosan have been proposed [48].

As shown in Figure 2, some methods exploit the chemical precipitation of chitosan at high pH to prepare chitosan flat structures. The chemical neutralization of chitosan by adding an alkaline solution to the polycationic electrolyte is mainly used for the synthesis of chitosan beads, particles, and fibers [49].

The immersion–precipitation phase inversion (IPPI) method [50] and the dry/wet phase separation (DWPS) process [51] have been proposed to fabricate flat ultrathin and defect-free asymmetric chitosan membranes for wound dressing applications. These methods combine the solution-cast and precipitation methods: in fact, a chitosan solution is cast on a support immersed in a coagulation bath (usually NaOH). Due to the mass transport of ions between the two phases chitosan precipitates in a porous flat structure.

Electrochemical deposition of chitosan [52,53] is another technique used to fabricate flat Chit films and coatings with highly controlled properties. Chit polycationic solution is used in an electrolytic cell to induce chitosan precipitation at the cathode, where the electrochemical reduction of water or molecular oxygen produces a local increase of pH at the electrode/electrolyte interface according to the following reactions:

2 H_2_O + 2e^−^ → H_2_ + 2 OH^−^ O_2_ + 2 H_2_O + 4e^−^ → 4 OH^−^

By regulating the physico-chemical properties of the solution, the nature of the electrodes and by controlling the applied current/potential and the deposition time, tailored self-standing flat chitosan structures can be obtained as detailed in several reviews [52,53].

The ionotropic gelation method has been utilized predominantly for the preparation of micro- and nanoparticles, synthesized by the addition of an anionic polyelectrolyte solution dropwise into an acidic chitosan solution. The anion polyelectrolyte solutions often contain alginate, carrageenan, xanthan, polyphosphates or organic sulfates [39,54,55]. Very recently phosphotungstates have been also successfully employed [9]. The ionotropic gelation method is discussed in depth in Section 4.2 for the synthesis of gel beads and in Section 4.3 for the synthesis of flat structures.

## 3. Applications of Chitosan Flat Structures

**Figure 4:**
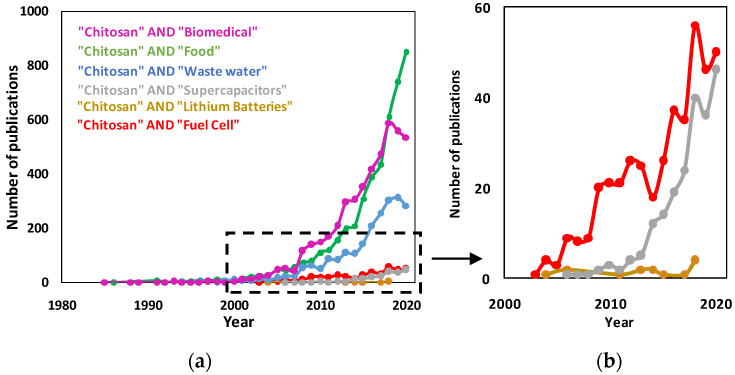
(**a**) Approximate number of publications from 1985 to 1st December 2020 obtained by Web of Science using the topic keywords indicated on the labels of the graph. (**b**) Magnification of the area indicated by the dashed box in the graph of panel (**a**).

Notwithstanding the fact that chitosan and some of its derived materials were discovered over two hundred years ago (1811), scientific research on their possible applications only started in 1983, when the *N*-deacetylation method to extract chitosan from chitin was proposed [56] and the number of related publications rapidly increased significantly in the 2000s. Flat chitosan structures (film, membranes, disks) have been mainly studied in terms of methods of synthesis, characterization and applicability in food packaging, waste water treatment and biomedical uses. In the last twenty years, the possible application of chitosan-based flat structures in energy conversion and storage devices has been also introduced and it is still under development. The analysis of the number of publications up to 1st December 2020 on the Web of Science database for various fields of application is reported in Figure 4.

In this section some example of applications of chitosan-based flat structures in biomedicine, energy conversion and storage, food and wastewater treatments/filtration are given.

### 3.1. Biomedicine

With the development of higher living standards in the world population, the need for improvement in the health and medical sector arises. Among the natural polymers, like cellulose, liver sugar, starch and chitin used in biomedical applications [57,58,59], chitosan has attracted attention because possesses antibacterial, antiviral, and anticancer effects. In addition, relevant chemical and physical modifications of chitosan give derivatives with desirable biodegradability, biocompatibility, bioactivity and non-toxicity properties [60,61,62,63,64]. Chitosan flat structures contribute to biomedical applications, especially for their ability to accelerate wound healing and optimize drug delivery. The incorporation into polymer blends is often employed to obtain composite polymers with antimicrobial properties and better thermal stability at the same time [65].

In targeted drug delivery chitosan derivatives are often preferred to protein drugs because of their unique structure and biological properties coming from their chemical structure and low cost [66,67]. A drug delivery technique introduces the right amount of drug to a specific place at the right time, improving the medical treatment and reducing the side effects of drugs. The drug delivery of proteins is not effective because proteins are easily degraded by enzymes. On the contrary, chitosan can easily deliver drugs to the specified place because its muco-adhesive, permeation, in situ gelling, and efflux pump- inhibitory properties [68]. Besides, Chit is the sole biodegradable polymer that possesses a cationic character among those reported in the various pharmacopoeias [69]. For instance, Almodovor et al. designed a LBL polyelectrolyte complex assembly using N-N trimethylchitosan as polycation and heparin as polyanion. This assembly was used for improving bone and allograft compatibility [70]. Sizílio et al. also synthesized by a solvent evaporation method a promising mucoadhesive chitosan membrane as a drug delivery system for betamethasone-17-valerate in the treatment of recurrent aphthous stomatitis [71].

**Figure 5:**
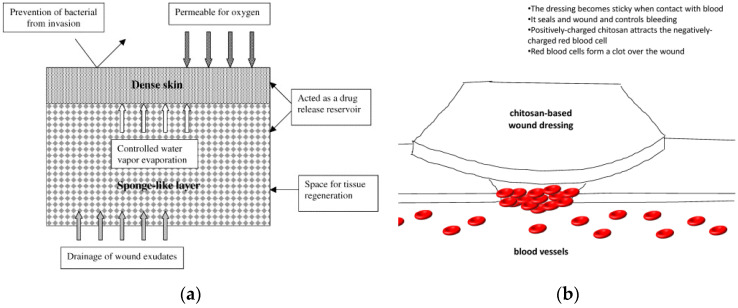
Sketch of (**a**) a flat chitosan-based asymmetric membrane for application in wound dressing. Reprinted from [51] with permission from Elsevier. (**b**) how the wound dressing works. Reprinted from [75] under open access CC-BY license.

Chitosan flat membranes have been also investigated for possible use as patches in tissue repair and regeneration. Chit polymer satisfies all the required properties for wound dressings: biocompatibility, non-toxicity, biodegradability, defined structure, good mechanical strength, high transport of gases, nutrients, proteins and cells [72]. A popular example of a flat chitosan membrane in wound dressing comes from the HemCon^®^ hemostatic bandage prepared by solution casting [73]. Furthermore, a novel concept of chitosan-based flexible membrane was proposed in 2017 [74] by using LBL synthesis on a patterned PDMS substrate. The membrane possesses a well-arranged porous structure to accommodate cell culture on one side, as shown in Figure 5a, and a dense layer on the other side that prevents bacterial invasion while allowing transpiration. This geometry allows high versatility and adaptability in the regeneration of multiple tissues, as shown in Figure 5b. Detailed updates on potential applications of chitosan flat structures in biomedical field can be found in [75,76,77,78,79,80].

### 3.2. Energy Storage and Conversion Devices

Fuel cells are environmentally-friendly electrochemical conversion devices with zero emissions [81,82,83,84,85] and are generally classified based on the electrolyte material used. This classification includes alkaline fuel cells, phosphoric acid fuel cells, proton exchange membrane fuel cells, molten carbonate fuel cells and biofuel cells [86,87]. Fuel cells possess high energy density, and they can operate without making noise [88,89]. However, the use of liquid electrolyte materials limits the deliverable performance, so the use of flat solid polymer electrolyte membranes is preferred to some extent. Polymer electrolyte membranes are required to possess good proton conductivity, resistance to electron conductivity and good mechanical and chemical strength in both dry and moisture state. The first polymer electrolyte membrane used in fuel cells was a sulfonated polystyrene membrane employed in the 1960s on the Apollo flight space missions as onboard power source [90]. Thereafter many commercially available membranes, such as Nafion^®^, Flemion^TM^ and Acipex^®^, have been used as polymeric ion conductive electrolytes [84]. However, Nafion^®^ and similar membranes are costly, and their ionic conductivity becomes insufficient without hydration at high temperatures. Among the materials studied to replace Nafion^®^ [91,92], chitosan has become popular in the last decade because of: (i) its environmentally-friendly nature and low cost [93], (ii) the presence of various functional groups that can be structurally modified to attain desirable and tunable properties [94] and (iii) its hydrophilic nature that makes it suitable for use at high temperature and low relative humidity. Pristine chitosan possesses very low proton conductivity, on the order of 10^−2^ S cm^−1^ at 25 °C, [95] that can be increased notably by relevant chemical reactions or physical assemblies involving the polymer functional groups [94,95,96,97].

This property has allowed the development of several methods for modifying chitosan for the obtainment of high-performance flat structures for fuel cells [97,98,99]. Many papers have reviewed the chemical modification of chitosan adopted to improve the proton conductivity of flat membranes: sulphonation [100], phosphorylation [101], quaternization [102] and chemical cross-linking [10,103]. The latter is commonly used because it guarantees higher chemical and mechanical stability of the modified polymer.

As detailed below, flat proton-conducting chitosan membranes have been fabricated typically by solution-cast [41,97,104,105,106] and chemical neutralization [107,108] techniques, while ionotropic gelation is a quite new method and still in evolution [9,95,96,109,110].

Nurulet al. [111] synthesized a cross-linked chitosan hydrogel for direct borohydride fuel cells. Chitosan solution was prepared by simply weighing the required amount of chitosan and mixing it with 2% (*wt*/*v*) glacial acetic acid solution in a beaker. This solution was magnetically stirred for 12 h to get a pale-yellow coloured chitosan solution that was allowed to dry in a Petri dish. The obtained solid chitosan was subsequently mixed with sodium sulphate and sodium hydrogen phosphate to obtain an ionically cross-linked chitosan hydrogel. The use of sodium sulphate and sodium hydrogen phosphate salts has the advantage of decreasing the cost of the whole device while maintaining the environmentally-friendly character of chitosan. The electrodes used in direct borohydride fuel cells are composed of Ni and C supported on palladium at the anode and C-supported platinum at the cathode. An ionically cross-linked chitosan hydrogel membrane was used as electrolyte cum separator. The performance of this fuel cell was reported to reach a maximum power density of about 800 mWcm^−2^ at 70 °C operational temperature. Feketeföldi et al. [112] used highly quaternized chitosan as anion conductor membrane in alkaline direct ethanol fuel cells. They prepared a quaternized chitosan membrane and quaternized poly(vinyl alcohol) membrane. The cross linking in these membranes was obtained by using glutaraldehyde and ethylene glycol diglycidyl ether as cross linkers. Equivalent blends of chitosan and polyvinyl alcohol membrane were prepared with different amount of cross-linking agent. The performance of this membrane in direct ethanol fuel cells showed that the membranes with lower cross linking have the best ionic and transport properties. Anion conductivities of 0.016 mScm^−1^ and ion exchange capacity of 1.75 meqg^−1^ were reported, whereas high cross-linking membranes showed reduced ethanol permeability of 3.30 × 10^−7^ cm^2^ s^−1^ at 60 °C. Layer by layer assembly has been also successfully used by Zhao et al. [48] to obtain Chit-phosphotungstic acid flat layers on SPAEK–COOH for application in direct methanol fuel cells. The resulting multi-layered membranes show methanol permeability values significantly lower than those relating to Nafion^®^ 117 membranes and great proton conductivity, required to guarantee direct methanol fuel cells with high power density. Cui et al. [41,42] prepared chitosan/phosphotungtic (SW), chitosan/phosphomolibdic (PMA) and chitosan/silicotungstic (SW) acid membranes by solution casting and subsequent functionalization by cross-linking. The methanol permeability values obtained for the three chitosan-based flat structures SWA, PWA and SiWA (respectively 2.7 × 10^−7^, 3.3 × 10^−7^ and 3.8 × 10^−7^ cm^2^s^−1^) decrease notably if compared with those of Nafion^®^ 117(2.4 × 10^−6^ cm^2^s^−1^). This result goes along with good values of proton conductivity. Xiong et al. [113] synthesized *N*-[(2-Hydroxy-3-trimethyl ammonium)propyl] chitosan chloride and they cross-linked it with glutaraldehyde. The cross-linked polymer was blended with chitosan to improve the performance of alkaline fuel cells in terms of mechanical strength, compactness and ionic transport within the electrolyte. Wan et al. studied the ionic properties of various chitosan membranes such as pure chitosan, chitosan in different deacetylation form, di-*o*-butyryl chitosan, glutaraldehyde cross-linked chitosan and they found that all of studied membranes were suitable for use as electrolyte in alkaline fuel cells [113,114,115,116,117]. Mat et al. designed a composite membrane that includes chitosan, PVA and CaO. Their studies showed that these flat composite membranes have good methanol barrier capability, a desirable property for use in direct methanol fuel cells [106].

Klotzbach et al. [118] designed novel chitosan membranes by modifying them with different aldehydes, such as butanal, octanal and hexanal. The resultant chitosan membranes are biocompatible and environmentally-friendly, being thus good candidates for replacing Nafion^®^ in sensor and fuel cell applications.

Novel chitosan/phosphotungstic acid proton-conducting flat membranes for low temperature H_2_/O_2_ fuel cells have been synthesized by ionotropic gelation of chitosan for the first time by Bocchetta et al. [110,119] and will be discussed in detail in Section 5.1.

Chitosan flat structures have been studied as electrolyte and/or electrode materials in different classes of batteries. Batteries are leading devices in energy storage technology because of their large-scale applications in portable devices and the electric vehicle industries. The anode material in lithium-ion batteries has been studied extensively. As an electrode material silicon shows good performance and a theoretical capacity of 4000 mA hg^−1^. It is important to select a suitable binder because it improves the cyclic performance of Li-ion batteries [120,121]. Polyvinylidene fluoride (PVDF) has been used in Li-ion batteries as a binder for Si anodes, but it did not provide good cycling stability because of its linear chain structure [122]. Lee et al. [123] used a cross-linked chitosan polymer as a water-soluble binder for Si anodes in Li-ion batteries. They prepared chitosan by dissolving 2 wt.% acetic acid aqueous solutions to obtain a 3 wt.% CS solution. After that glutaraldehyde was added to the chitosan solution to get the cross-linked chitosan. A homogeneous slurry was prepared by mixing Si powder, super P and chitosan in a weight ratio of 60:20:20 in acetic acid. This slurry was then spread over a copper foil and allowed to dry to obtain a chitosan binder Si anode. The cross-linking glutaraldehyde firmly bound the chitosan and Si. This cross-linked binding improved the electrochemical performance of the battery. The chitosan-based Si anode showed the discharge capacity of 2782 mAhg^−1^ with a high initial columbic efficiency of 89% and capacity of 1969 mAhg^−1^ at the current density of 500 mAg^−1^ over 100 cycles.

Lithium sulphur batteries are a class of batteries that are attracting attention because of their high theoretical specific capacity (1675 mAhg^−1^), better energy density and the high abundance of sulphur. These batteries are limited by factors such as expansion of sulphur during the charge/discharge process and insulating nature of S/Li_2_S. Using suitable binders for electrode material can minimize these limitations of lithium-sulphur batteries. Bio-based polymers such as chitosan are a good option as a binder because of their eco-friendly nature. Lee et al. synthesized [124] a new multifunctional binder which is composed of reduced graphene oxide (rGO) and chitosan. The chitosan binder enhanced the capacitance of Li-S batteries. The binder was prepared by a modified Hummer’s method by simply mixing chitosan and graphene oxide in aqueous solution [125]. This mixture was then subjected to heat treatment at 90 °C for 6 h. Without the heat treatment chitosan-GO binder showed poor electronic conductivity. The electronic conductivity of chitosan-rGO binder was 11.4 times higher than chitosan-GO binder without heat treatment. The chitosan-rGO binder improved the capacity decay retention of 0.016% per cycle at 1 C over 1000 cycles. The battery also showed stable capacitive performance and the shuttle effect was reduced by interactions of functional groups of chitosan polymers. The integration of rGO in chitosan gave firmness in the structure and conductivity also enhanced. This mechanical strength provided by binder reduced the sulphur volume expansion to some extent.

Si electrode materials have high energy density as compared to conventional graphite electrodes. Abu Labdeh et al. [126] fabricated a Si and graphite composite electrode using a cross-linked chitosan polymer. The Si/graphite composite was made by simply mixing nanosilicon powder, graphite, and super P black (as a conductive agent). Chitosan was also added to the mixture as a binder. The slurry of the mixture was then coated on a copper foil and dried in the air at 85 °C. The final product was used as an electrode in Li-ion batteries. The effect of acetic acid on the performance of Si/graphite electrodes with chitosan binder was studied. They found that 30 wt.% or more acetic acid stabilized the cycling performance of batteries at 50 cycles. They were the first to use the carboxylic acids in chitosan cross-linked binder for Si/graphite composite electrodes for Li-ion batteries. The performance of these materials is excellent combined with benefits of low cost and eco-friendly nature. These advantageous properties make chitosan binder useful in other electrode materials that undergo a volume expansion during charging/discharging of Li-ion batteries.

Sodium ion batteries could be a better alternative to Li-ion batteries because elemental sodium is more abundant in Nature and the electrochemical redox potential of sodium is lower than that of lithium. However, the search for suitable anode materials for sodium ion batteries is still a challenge. Goodenough et al. [127] prepared an antimony anode using a cross-linked chitosan polymer as a binder in Na-ion batteries. When a chitosan cross-linked antimony electrode is prepared, the chitosan and glutaraldehyde are not mixed first because they react rapidly and the required fluidity is not achieved for chitosan. Sb particles and carbon black were first mixed with chitosan solution and after that glutaraldehyde was added as cross-linking agent to obtain the slurry mixture. The slurry was then coated on a copper foil and dried to get a uniformly distributed C-black and chitosan-bound antimony flat electrode. The performance of the Sb electrode was tested using coin cells of these electrodes and Na ion as counter electrode. The electrolyte NaClO_4_ in propylene carbonate electrolyte was used with a fluoroethylene carbonate additive. Chitosan binder provided stable cyclic battery performance and a charge capacity of 555.4 mAhg^−1^ at 1 C after 1000 cycles. The capacity retention was found to be 96.5% compared to the initial cycle. The designed flat cross-linked chitosan polymer effectively reduced the large volume change of the antimony anode upon sodiation and de-sodiation processes.

Lithium-sulphur batteries are considered the next generation energy storage devices because of their high theoretical capacity, high energy density. In addition, sulphur is cheaper, highly abundant, and eco-friendly [128,129,130,131]. The polysulfide shuttle effect is one of the main problems of lithium-sulphur batteries. Wang et al. [132] designed a cathode synthesis based on molybdenum disulphide-coated nitrogen-doped mesoporous carbon spheres/sulphur (NMCS@MoS_2_/S) and a carbon nanotube (CNT) and chitosan-based flat separator. Earlier studies showed that with high sulphur loading it was difficult to achieve high specific capacity and good cyclic stability. In this study, they proposed novel NMCS@MoS_2_/S cathode and chitosan-based separator materials able to increase the cyclic stability and rate performance. The NMCS@MoS/S and CNT-chitosan battery showed high capacity of 893 mAhg^−1^ with decay in capacity of 0.04% after 200 cycles. The reversible capacity was reported as 827 mAhg^−1^ with a capacity retention of 92.4% at 0.5 C after 200 cycles.

Supercapacitors (SCs) are energy storage devices showing higher power density as compared to batteries and conventional capacitors and find application in hybrid electric vehicles, load leveling, military and medical devices. The high-power density of supercapacitor is due to large surface area of electrodes and porosity of electrode material. Based on the electrode material, SCs can be classified into electrochemical double layer capacitors (EDLCs), pseudocapacitors and hybrid supercapacitors. In EDLCs, the electrode is typically made of carbon and the electrolyte can be either aqueous or non-aqueous in liquid or solid state.

Chaudhary et al. [133] prepared a cross-linked chitosan hydrogel membrane electrolyte by a solution casting technique by using sodium sulphate as cross-linking agent. The prepared ionically cross-linked chitosan hydrogel membrane electrolyte was used as both electrolyte and separator. Zhang et al. [134] synthesized a porous carbon by blending gelatin and chitosan in acetic acid solution through a facile method. The blended product was treated with KHCO_3_ to improve the morphology of the carbon material. The specific surface area of this novel porous carbon was reported to be 927.17 m^2^g^−1^. The supercapacitor showed a good pseudocapacitive behavior. A specific capacitance of 331 Fg^−1^ in 6 mol/L KOH electrolyte at 1 Ag^−1^ was reported and capacity stability of 90% after 1000 cycles at 10 Ag^−1^ was retained. The energy density of 34 WhKg^−1^ was greater than many commercial devices.

**Figure 6:**
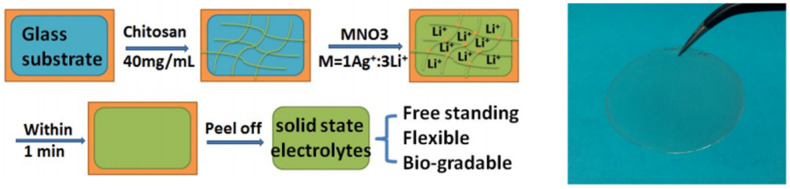
Synthesis of a flat-structured free-standing solid polymer superamolecular hydrogel electrolyte. Reprinted under an open access CC-BY licence [135].

Novel supramolecular hydrogel-type solid state electrolytes (SHEs) with excellent supercapacitor performance have been also prepared by functionalization of chitosan–Ag^+^ hydrogels with lithium ions [135]. Chitosan–Ag^+^ hydrogels are synthesized via cross-linking by supramolecular complexation between chitosan, and Ag+. As shown in Figure 6, Chit solution is placed on a flat glass and a mixed AgNO_3_ and LiNO3 aqueous solution was added on the top. After 1 min the SHE is formed and can be easily peeled off the substrate. Cyclic voltammetry technique reveals that the supercapacitor with a two-electrode configuration containing the SHE membranes possesses a quite ideal electrochemical double layer capacitive behavior and fast charge/discharge properties. Also, the capacitance at a current density of 1.8 mA cm^−2^ is 10 mF cm^−2^ and after 10,000 consecutive charge–discharge cycles, the coulombic efficiency was still nearly 99%.

### 3.3. Food Packaging

Applications of chitosan flat structures in eligible and environmental fields are the oldest [33,136,137]. Food storage and food management required the use of synthetic chemicals; thus, some new non-toxic natural compounds should be explored for these applications. Chitin and chitosan have proven themselves as useful materials in this area because of their attractive antimicrobial, color stabilizer, flavour extender and beverages additive properties [138,139]. More precisely, chitosan membranes are the best option for food industry applications. These membranes have moderate water permeability and lower permeability to oxygen, nitrogen, and carbon dioxide than other available membranes [140]. Apart from being a good antimicrobial and antifungal material, chitosan provides better mechanical strength, which allows excellent wrapping, packaging, and coating materials for food objects. Simply dipping foods in chitosan solutions can easily coat the food material. The conseration time of these food materials increases by using a chitosan coating.

A noted effect of chitosan coatings is the delay in the enzymatic browning of vegetables and fruits. For example, Ahmad et al. [141] prepared apple peel nanoparticles from apple peel and then these nanoparticles were added into chitosan and gelatin-based packaging films. SEM results revealed that as we increase the concentration of apple peel ethanoic extract (APEE), sintering of the nanoparticle film takes place. The presence of APEE in the chitosan/gelatin improved the physical properties of the film by increasing the thickness. Other properties such as solubility and water permeability were decreased by the APEE presence.

### 3.4. Wastewater Treatment and Air Filtration

Chitosan has been used in wastewater treatment for over 30 years because its excellent coagulation, flocculation and metal chelation properties due to the high number of amino and hydroxyl groups in the chemical structure of Chit [142,143]. Solids contained in food processing wastes and dyes coming from textile industry can be removed by taking advantage of the coagulation and flocculation properties of chitosan. The adsorption features of chitosan can remove toxic materials from plastic and lubricants and polychlorinated biphenyls as well. Phenol, a waste from the pulp and paper industry, can be converted into *o*-quinone by the enzyme tyrosinase and then removed by reaction with the amino groups of chitosan [144,145,146]. Thanks to the chemical properties and the high porosity [147,148] chitosan exceeds the heavy metal binding capability of activated carbons by a big margin. Different processes can achieve the decomposition of heavy metals. Chitosan-based powders and gel beads have been used for decontamination purposes [149]. Another approach is the ultrafiltration of metal chitosan complexes prepared in solution before filtration [150]. Finally, highly Hg(II)- heavy metal selective chitosan flat membranes can be prepared by immobilizing a dye, procion brown MX 5BR, on pHEMA/chitosan composite membranes [151].

**Figure 7:**
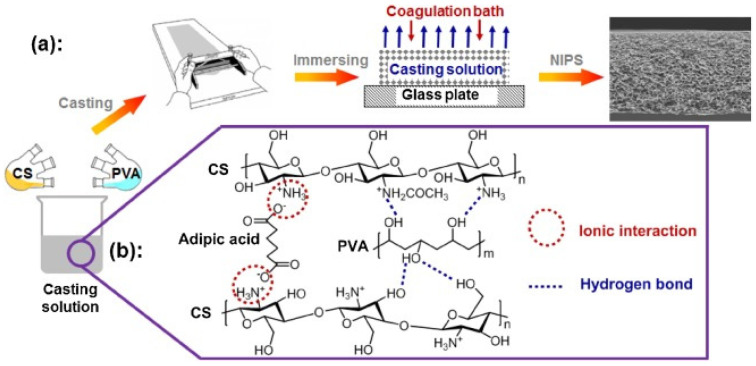
(**a**) Schematic of preparation process of Chit/PVA blend membranes. (**b**) Proposed interactions among Chit, PVA and adipic acid in the casting solution. Reprinted from [152] with permission from Elsevier.

Antibacterial and environmentally friendly chitosan-based flat membranes have been also investigated for air filtration (see a collection of papers in [142]) to prevent environmental and human body pollution, especially by particulate matter (PM). PM has a long lifetime and adsorbs toxic compounds and bacteria in the air due to the larger surface areas, and can penetrate into the respiratory system and even the blood. In Figure 7, we report the scheme of synthesis of Chit/polyvinyl alcohol blend flat membranes for air filtration [152].

## 4. Mechanisms of Ionotropic Gelation

### 4.1. Fundamentals of Ionotropic Gelation

Ionotropic gelation (IG) is a phenomenon in which polyelectrolytes (PEs) come in contact with oppositely charged small molecules or macromolecules causing a liquid-gel phase separation, with the formation of a polymer-rich phase (gel) and a polymer-poor phase (liquid) surrounding the former [1]. The process is strictly governed by the experimental variables of the buffer medium, such as pH or ionic strength and the physico-chemical composition of the polyelectrolyte. This method is usually employed for the synthesis of natural water-soluble polymeric nanoparticles with a high control in the release of bioactive materials by polymer relaxation. The hydrogel beads are synthesized by addition of drug-loaded polymeric solution drops to the aqueous solution containing a cationic polyelectrolyte [153,154,155]. Polymers usually involved in these processes are natural, hydrophilic and biodegradable ones, such as sodium alginate [156], gelatin [157], carboxymethyl cellulose [158,159] and chitosan. Synthetic polymers can be also used (Table 1).

### 4.2. Ionotropic Gelation for the Synthesis of Nano/Microparticles

Natural, biocompatible, and biodegradable polymers, such as chitosan, gelatin and alginate are commonly used in ionotropic gelation for biomedical applications [161]. The classical procedure involves electrostatic interaction between two ionic species of opposite charge to produce polymeric nano/microparticles. In the case of chitosan, its amino groups are positively charged in dilute acid solution, allowing the interaction with anionic species [1]. Room temperature, stable stirring, appropriate concentration of counterions, and a constant mixing rate are among the conditions that mediate the spontaneous formation of the particles [162,163].

Chitosan and sodium tripolyphosphate (TPP) are the most studied and the most used ionic pairs for ionotropic gelation [8]. In drug-delivery approaches, biologically active molecules are added to the reaction, becoming trapped within the physical network formed by ionic species. Additional charged groups also play a role, for example: protein encapsulation takes advantage of the presence of aminoacids with positive and negative charges, which can interact with any of the counterions. Considering a model system with chitosan and TPP, basic amino acids with an extra amino group interact with the highly electronegative phosphate groups of TPP, while acidic amino acids can be attracted to positively charged chitosan chains. This has allowed the encapsulation of bovine serum albumin (BSA) [164,165], interferon alpha [4], insulin [166], and cationic peptides [167] with high encapsulation efficiency in chitosan-TPP nanoparticles.

Several studies have been conducted to improve drug performance in terms of extended bioavailability and stability, which have demonstrated the potential impact of ionotropic gelation in biomedicine. The cellular uptake of molecules encapsulated in 200 nm chitosan nanoparticles, at 24 h, was observed in the case of the anticancer agent resveratrol [168] and BSA [3]. Similar in vivo results in a single-pass intestinal perfusion model were observed for puerarin (a molecule with bioprotective activity and obtained from a leguminous plant), which increased its bioavailability by a factor of 4.4 when encapsulated in chitosan nanoparticles [169]. Other approaches include the synthesis of thermo-responsive structures [2] and micro/nanoparticles [170,171] encapsulating the chemotherapeutic (anticancer agent) doxorubicin; and the combination of a chemotherapy (5-fluorouracil) with magnetic nanoparticles to obtain a core-shell structure for drug delivery and hyperthermia therapy [172].

Ionotropic gelation of chitosan has also been performed with other anionic agents, especially in combination with alginate [173]. This process is considered to increase particle stability by the addition of a second polymeric protective layer. The classic procedure is slightly modified by a first step (pre-gelation) in which one of the polymers interacts with a small ionic agent (metal salt), and a second phase when the remaining polymer is added (polyelectrolyte complexation). In this manner, physically cross-linked chitosan/alginate nanoparticles have been synthesized for the encapsulation of different bioactive molecules, including insulin [174,175].

### 4.3. Advantages and Disadvantages of Ionotropic Gelation

The ionotropic gelation method has many advantages [176,177,178] over other formulation tecniques. Simplicity and low cost are the most important and are related to the absence of sophisticated equipments, acqueous solvents and to the short times of the process. In addition, the reversibility of the physical cross-linking by electrostatic interaction, instead of chemical crosslinking, allows to avoid possible toxicity and other undesirable effects for biomedical application. Also, an elevated encapsulation efficiency can be reached when the process is designed to achieve optimal polymer-drug interactions [4].

Many years ago, the main disadvantage of ionotropic gelled polymers was their low mechanical stability [179,180]. However, today many solutions have been developed to produce mechanically stable polymers by ionotropic gelation. Particularly, the formation of polyelectrolyte complexes between chitosan and other polymers such as alginate, dextran, chondroitin [178,180], and the modification of chitosan chains with carboxyalkyl and other functional groups [181]. This has lead to a wide range of functionalization possibilities and to increased ionic interactions, which favor continuous improvement and future applications of the ionotropic gelation technique.

### 4.4. Ionotropic Gelation of Chitosan

In the case of chitosan, simple or complex coacervation events can—depending on the number of macromolecules involved in the process—be achieved by exploiting the cationic nature of this unique biopolymer [181]. In essence, chitosan behaves as a polycation for pH values of buffer medium <6, thereby resulting prone to interact with negatively charged polyions [11,182]. In this sense, the pKa of chitosan was determined in the range 6.3–7.2 depending on the fraction of acetylated units (FA) of chitosan, [183] although other findings reported no significant dependence of pKa on FA [184,185].

Multivalent anions such as tripolyphosphate (TPP) or pyrophosphate (PPI), and polyanions as well as hyaluronate or alginate are commonly used to foster electrostatic interactions with chitosan [186,187,188], thus promoting a true “ionotropic gelation” event. The ionotropic gelation of chitosan has been widely explored over the last years to assemble micro- and nanocolloids, especially for drug delivery applications [189]. Since biological fate of these systems is strictly correlated with the colloid dimension, shape, rigidity and homogeneity [190,191,192] controlling the gelation conditions is of pivotal importance. Beside precise stoichiometry between chitosan and negatively charged linkers, it has been recently recognized that in the case of simple chitosan-TPP ionotropic gelation the mixing method plays a cardinal role in modulating physical properties of resulting colloids [193,194].

On the other hand, few attempts have been undertaken to fabricate macroscopic chitosan gels by exploiting ionotropic gelation methods. Contrary to the reticulation without involving any type of cross-linker [195,196,197], chitosan ionic gelation represents a more demanding approach due to fast self-assembly of the biopolymer in the presence of ionic cross-linkers, causing severe inhomogeneity of resulting gels at the macro-scale.

Three-dimensional structures of chitosan have been recently obtained by ionic gelation of chitosan with 6-phosphogluconictrisodium salt (6-PG-Na^+^) [198]. This set of gels are interesting from a biological point of view, since they were found to be no toxic and dermal bio-compatible, showing potential application in topical administration and wound dressing. Another example comes from Vårum and co-workers, who developed ionic chitosan gels through mannuronan oligomers as cross-linker and a neutral soluble chitosan showing fraction of acetylated units, FA, of 0.4 [199]. Ionic chitosan gels reticulation can be also governed by electrostatic interaction between a metal polyanion, such as molibdate (Mo_7_O_2_^6−^) [200] or phosphotungstate (PW_12_O_40_)^3−^ [9], and Chit amine groups under acidic conditions.

## 5. Ionotropic Gelation of Chitosan Flat Structures

**Figure 8:**
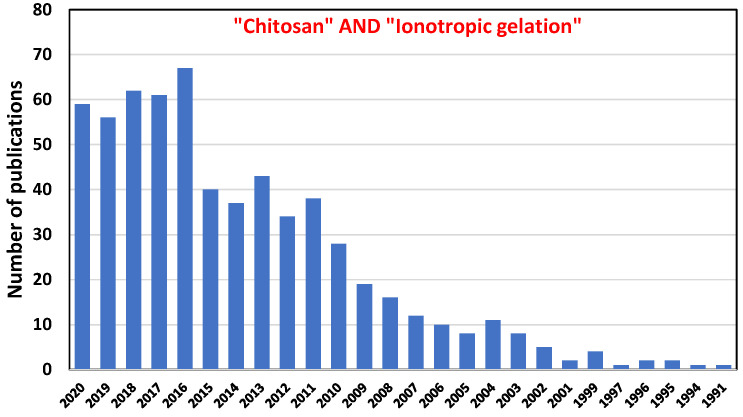
Published articles each year obtained by a search in Web of Science database in December 2020 using “Chitosan” and “Ionotropic gelation” topic keywords.

According to our literature search, the ionotropic gelation of chitosan spherical particles was observed for the first time in 1981 [201]. The ability of the Chit particles to entrap *E. coli* cells in an easy and cheap method with sterile conditions generated an increase of the research interest of this method especially for biomedical application (Figure 8).

The use of ionotropic gelation for the synthesis of flat structures is quite new and finds application in energy conversion devices (Section 5.1) and biomedicine (Section 5.2).

### 5.1. Ionotropic Gelation of Chit Flat Structures through Porous Alumina Support

**Figure 9:**
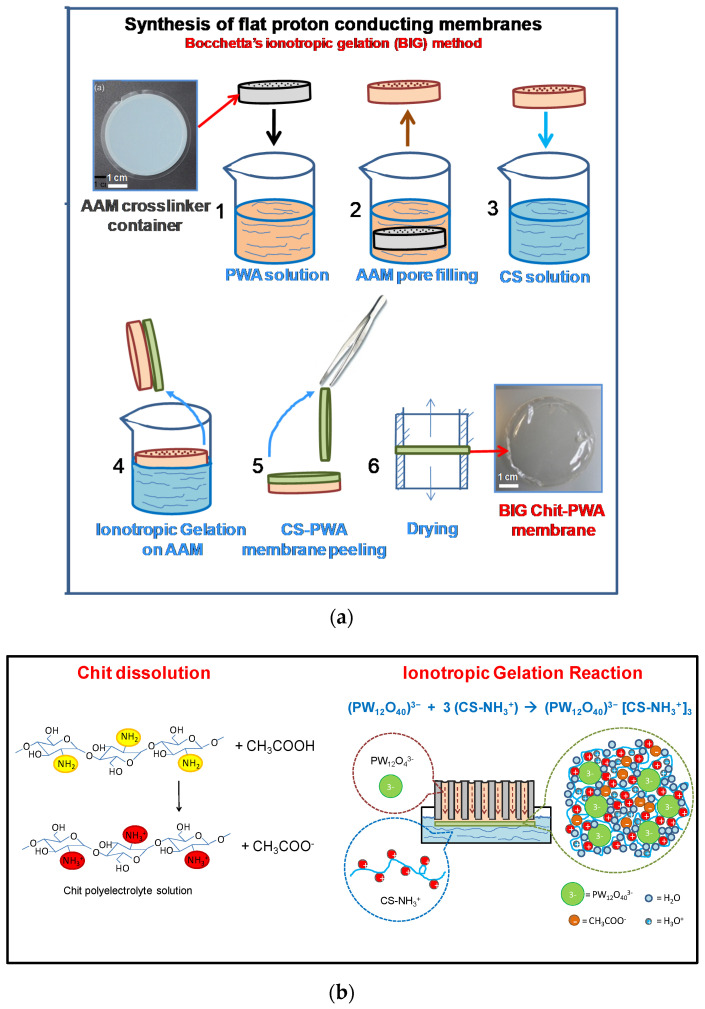
(**a**) The BIG method: steps of the procedure of proton conducting Chit-PWA flat membranes formation. (**b**) Scheme of Chit polyelectrolyte dissolution in acid acetic solution (left) and ionotropic gelation of chitosan through cross-linking with PWA^3−^ anions (right). Reproduced from Ref. [9] under open access CC-BY license.

A novel ionotropic gelation method [9] has been recently proposed to synthesize proton-conducting flat chitosan membranes for application in room temperature H_2_-O_2_ fuel cells by using renewable sources (Figure 9a). According to this approach, ionotropic gelation of chitosan is performed by using a porous anodic alumina (AAM) support to organize the formation of flat structures as well as to produce highly proton conducting electrolytes. In a single process the simultaneous formation of three-dimensional polymeric gel structures and proton conductive sites has been obtained thanks to the use of a proton conductor ionic cross-linker. Ionotropic gelation confers to the polymer proton conductivity in a more uniform way with respect to functionalization step after the chitosan membrane synthesis. In fact, solution-cast pre-formed chitosan membranes subjected to functionalization show lower proton conductivity due to slow surface-to-bulk diffusion of functional molecules through the polymer.

The BIG process is based on the use of AAM support, which shows uniform arrays of cylindrical pores containing the functional solution [109,202,203,204] that is released in a controlled way with respect to a totally opened surface. The ionotropic gelation process occurs at one side of the AAM support, where the ionic cross-linker solution covering the surface is exposed to the Chit solution (Figure 9b) allowing the growth of chitosan flat structures.

In Figure 9 the experimental steps followed to prepare ionotropic gelled chitosan through AAM are detailed. First, a solution containing a cross-linker (in this case phosphotungstate polyanions) is prepared (step 1). In step 2 the pores of the AAM support are totally filled with the cross-linker solution by immersion for established times. Then, chitosan is dissolved in slightly acidic solution (see the protonation reaction illustrated in Figure 2) to form a polycationic electrolyte (step 3).

Step 4 shows the ionotropic gelation reaction: one side of the AAM support containing phosphotungstic acid solution is put in contact with the chitosan solution. Positively charged –NH_3_^+^ groups of chitosan react with negatively charged phosphotungstatepolyoxy-anions (PW_12_O_40_)^3−^ at the AAM/Chit solution interface forming a functionalized chitosan polymeric flat structure. The gel polymeric network formed by ionic cross-linking is slightly adherent to the AAM substrate and can be easily peeled off the AAM by using tweezers (step 5) for subsequent drying.

Using a multi-technique approach the evolution of the ionotropic process with time can be monitored. For example, the gelation rate of chitosan in the BIG process (shown in step 4 of Figure 9) has been studied by SEM, EDX, FTIR, DTA and analysis in a H_2_/O_2_ fuel cell.

Thickness measurements, obtained for example through SEM techniques, can address the understanding of the kinetics of the gelation process. In [9], it has been found that the gelation kinetics are very fast in the first instants of AAM immersion in the Chit solution because the electrostatic nature of the bonds forming between PWA^3−^ and Chit-NH_3_^+^ at the AAM/Chit sol interface (PWA 0.76 M, 2% *wt/v* chitosan). Afterwards a kinetic slowdown is recorded due to the diffusion of PWA anions into the previous formed chitosan until the maximum thickness is formed. The results reported in [9,110,120] allowed hypothesizing a kinetic control governed by the phosphotungstate ions through the Chit layer formed in the first instants at the AAM/Chit solution interface. The diffusion of PWA solution into a gel of flat geometry can be controlled by two different mechanisms: (i) the concentration gradient and (ii) the polymer relaxation phenomena [65]. By using a semi-empirical approach derived from the one-dimensional Fick’s second law of diffusion applied to chitosan [63], it has been found that at the experimental conditions used in the paper the penetration rate of PWA solution is lower than that of the polymer chains relaxation [9]. At those conditions (0.76 M PWA, Chit 2% *wt/v*), chitosan is the limiting reagent of the ionotropic gelation reaction and the thickness of the flat chitosan structures grows linearly with the concentration of Chit solution [110]. Chitosan films with poor mechanical strength not able to self-stand after peeling are obtained by using stoichiometric concentrations of PWA solution (~0.05 M PWA for Chit 2% *wt/v*). This means that that not all the PWA^3−^ anions s available for the crosslinking reaction, which is fundamental for the construction of well-structured three-dimensional polymer film [59]. EDX analysis performed along the cross-section Chit-PWA (Figure 4a,b) can be used to give count of the PWA^3−^ amount in the polymer film. Measurements of the W/C atomic ratio at different times (from 2 to 60 min) of the ionotropic gelation process show a slight increase in the first minutes accounting of the PWA solution diffusion through thinner Chit-PWA film followed by a quite constant trend. The results suggest that a complete crosslinking of the chitosan gel during ionotropic gelation can be favored by a slower diffusion of the PWA anions in the polymer. In fact, by performing post-crosslinking treatments of the as-formed ionotropic gelled Chit-PWA, a further increase in crosslinking grade is measured by EDX and confirmed by DTA and FTIR analysis [9].

**Figure 10:**
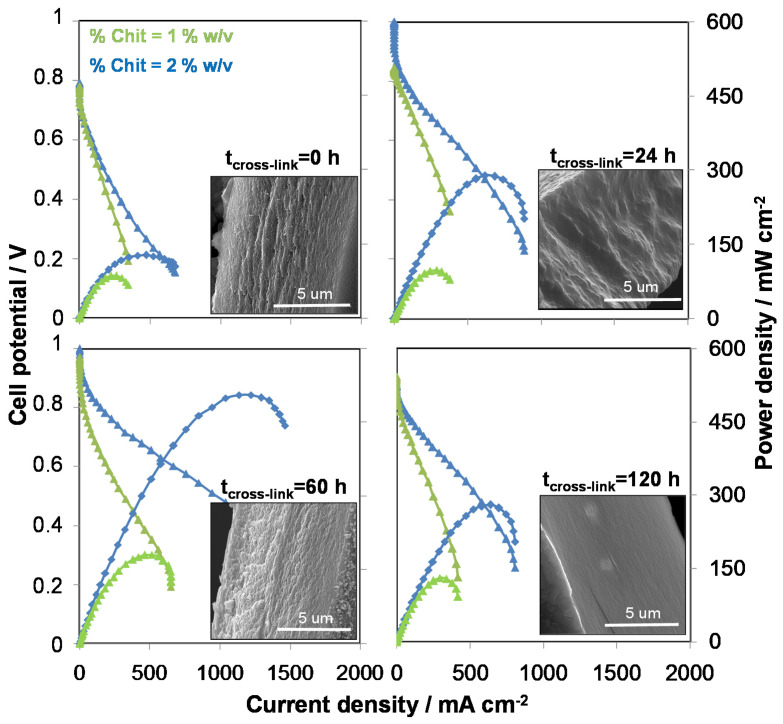
Fuel cell performance recorded for a hydrogen/oxygen fed fuel cell (25 °C, 1 mg cm^−2^ Pt) with Chit/PWA flat membranes prepared by ionotropic gelation at the experimental conditions indicated on the graph. Insets: SEM cross-sections of inotropic gelled Chit/PWA flat membranes before assembly. Reproduced from Ref. [9] under open access CC-BY license.

For applications in energy conversion devices, such as fuel cells, the obtainment of the right PWA content in the flat Chit-PWA membranes is crucial because it determines their proton conductivity. A detailed analysis of their behaviour in a room temperature H_2_/O_2_ fuel cell has been conducted by recording the polarization curves and power output of Chit-PWA membranes prepared by the BIG methods in different conditions [9,110,120]. In Figure 10 the results obtained for Chit-PWA membranes prepared at two chitosan concentrations and four post-crosslinking times are reported. As expected, the fuel cell performance increases until the ionotropic gelation reaction is completed, and all the protonic sites of chitosan have been cross-linked with phosphotungstate anions. The proton conductivity data of the flat chitosan membranes obtained by in-situ electrochemical impedance spectroscopy (EIS) at open circuit conditions in the fuel cell are in good agreement with those calculated by fitting the fuel cell polarization curves in the ohmic region [9,110,120].

To conclude, ionotropic gelled Chit-PWA membranes provide a maximum power density in H_2_/O_2_ fuel cell (~500 mW cm^−2^) and the proton conductivity (~1.5 10^−2^ S/cm) of Chit-PWA membranes prepared by the BIG method are values of interest in the literature scenario of bio-based proton conductive membranes [3,34,37,42,43,44,45,46]. Besides, the possibility to increase the PWA content in order to further enhance the fuel cell power output is the next challenge of this ionotropic gelation method.

### 5.2. Ionotropic Gelation through Slow Diffusion of the Cross-Linker

**Figure 11:**
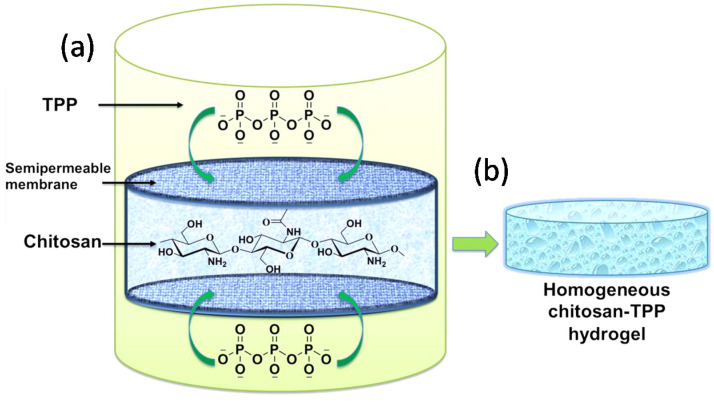
(**a**) Experimental setup for developing chitosan-tripolyphosphate (TPP) macroscopic gels through external gelation method. (**b**) Scheme of the obtained homogeneous chitosan-TPP hydrogel. Reprinted with permission from ([13], 2014 American Chemical Society).

Recently, Sacco and co-workers developed an experimental setup for the fabrication of ionic chitosan gels harnessing a controlled diffusion of tripolyphosphate (TPP)—used as multivalent anion—from an outer incubation medium toward a confined polymer solution (Figure 11) [13,36]. More in detail, chitosan is acid solubilized to protonate all glucosamine units, cast into a mold, closed by two dialysis membranes, and fixed by double circular stainless iron rings. The system is hermetically sealed and immersed into a gelling solution containing TPP. While the chitosan concentration is typically set at 3% *w*/*v*, the amount of TPP in the gelling solution can be varied based on the chitosan chemical composition, i.e., the fraction of acetylated units (FA), thereby the molar ratio (r) between TPP and repeating unit of chitosan (r = [TPP]/[chitosan]r.u.) ranges from 5.2 to 3.8 at the beginning of the process [8]. It is important to stress that no macroscopic gel formation can occur for r <3.1, as rather heterogeneous aggregates develop. The pH of gelling solution is set at 4.5 and ion diffusion proceeds slowly for 24 h under moderate stirring at room temperature allowing for gel formation. It should be remarked that only a moderate amount of TPP(~13%) was found to diffuse within chitosan solution during 24 h incubation, but at same time adequate for promoting a true (macroscopic) ionotropic gelation event [13]. Furthermore, ^31^P-NMR analyses demonstrated that TPP diffusion was faster in the first 4 h of incubation, while flattening up to 24 h, clearly indicating that the uptake of the cross-linker is temporally dependent and not immediate, thus fostering the assembly of a well-defined wall-to-wall3D network rather than irregular macro-aggregates.

The mechanism underlying chitosan macroscopic gelation is supposed to include three consecutive steps: (i) the formation of small primary complexes as already known in the synthesis process of Chit micro-gel [205]; (ii) the consequent aggregation into bigger micro-gels of higher-order; (iii) the aggregation of micro-gels through both electrostatic and van der Waals bonds among polymer chains [8].

**Figure 12:**
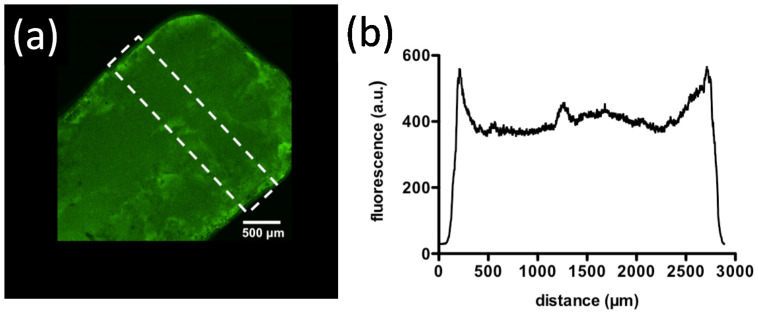
(**a**) Confocal scanning electron microscopy on chitosan gel synthetized by FITC-labeled chitosan along with (**b**) polymer profile through the gelling axis. Reprinted under an open access CC-BY licence [207].

Supporting salts like NaCl can be eventually added in the process in order to improve the gel homogeneity when low chitosan amounts are tested (i.e., 1.5% *w*/*v*). The presence of NaCl is necessary at low chitosan concentration - hence low viscosity of solution -to shield strong electrostatic interactions between the polymer and TPP, hence reducing binding kinetics. The overall effect is to limit chain diffusion toward the TPP-gel interface and, consequently, formation of a polymer gradient. If the latter occurs (no added NaCl and low chitosan viscosity as experimental conditions), chitosan progressively accumulates forming a gelling zone, resulting into an inhomogeneous network with the highest concentration of polymer at the boundary and a liquid-like core [13] in agreement with similar ionic gelling systems [206]. The formation of polymer gradient has been corroborated at a later time by means of confocal microscopy analyses, using a low molecular weight chitosan ($\bar{M_{V}}$ = 120,000) as model (Figure 12) [207]. To conclude, while high molecular weight chitosan’s (or high concentrated solutions) assemble homogeneous networks by hampering the easy migration of polymer chains toward the TPP-gel interface due to high viscosity, low molecular weight counterparts or low concentrated solutions generate more inhomogeneous systems, where in the latter case supporting salts such as NaCl are needed to form 3D continuous networks. Overall, the present experimental setup avoids the standard instantaneous ionotropic gelation of chitosan when oppositely charged polyions such as TPP are mixed, permitting a uniform distribution of the cross-linker throughout chitosan solution.

**Figure 13:**
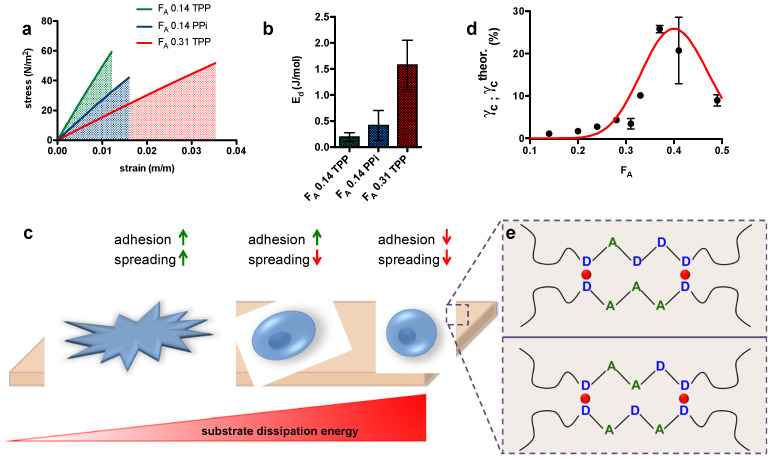
Substrate dissipation energy as novel cell controller. (**a**) Stress as a function of strain for different chitosan substrates. (**b**) Energy at different fractions of acetylation (F_A_). (**c**) How the substrate dissipated energy controls adhesion and spreading. (**d**) Critical strain as a function of F_A_. (**e**) Scheme showing how chitosan substrate allow two possible combinations of five consecutive monomers (pentads) irrespective of sugar position behave as “energy dampers” thus dissipating shear forces (A = N-acetyl-glucosamine unit, D = glucosamine unit). Reproduced from [209] with permission of John Wiley and Sons.

Recently, this set of biomaterials have been studied as cell substrates for tissue engineering applications and, more broadly speaking, for the investigation of cell-to-extracellular matrix interplay [208,209] Along the two research lines, the study of mechanical properties plays a cardinal role to consider ionic chitosan gels as promising extracellular matrix mimics. The authors of the same research group demonstrated that gel mechanics can be finely modulated by varying the polymer, TPP and NaCl concentration, [13] type of cross-linker, [8] and polymer features such as molecular weight and chemical composition [207]. For instance, it was found that gel stiffness and linear elongation augmented with increasing chitosan molecular weight and degree of acetylation, respectively [207]. From the biological viewpoint, chitosan-based gels did not show cytotoxicity in vitro after conditioning in phosphate buffered saline (PBS) buffer, and represent natural substrates that allow cell anchoring [208]. Strikingly, findings on how cells probe and integrate extracellular matrix mechanics have been unveiled only very recently using ionic chitosan gels as cell substrates [209]. Specifically, chitosan-based viscoelastic substrates endowed with different inherent dissipation energies have been developed to study cell adhesion and spreading. By combining gel stiffness and linear elongation into a singular parameter, the authors introduced a molar energy (J/mol) contribution required to deviate from linear stress-strain regime and enter the strain-softening (plastic) region. By progressively increasing substrate dissipation energy, they proved that cells lose the ability to spread and adhere (Figure 13). This contribution pointed at the combination of facing sugar sequences composed of five monomers (pentads) as “dampers” in dissipating shear forces. Although hypothesized previously [210,211], the present study quantify the dissipation energy term linked to extracellular matrix, and lays the foundations of novel interpretations about cell-to-substrate interplay.

Lastly, chitosan gels can be freeze-dried to generate pliable membranes incorporated with silver nanoparticles [212]. These constructs proved noteworthy antibacterial properties showing at the same time good safety, thus representing promising candidates for the treatment of non-healing wounds.

## 6. Conclusions

Over the last years the ionotropic gelation of chitosan has been widely exploited for the preparation of nano- and micro-gels especially employed in the biomedical sector. Chitosan-based flat structures assembled through ionotropic gelation are gaining significant interest among scientists, since potential applications thereof encompass ones ranging from wastewater treatment to energy conversion and storage to mention but a few. Here, we have provided the actual state of the art on this hot matter explored in a very limited way hitherto. From this perspective, ionotropic gelled chitosan-based flat structures emerge as intriguing networks that deserve to be further investigated allowing simplicity, low cost and physico-chemical tunability as a function of the required application. The numerous references discussed in the paper provide a comprehensive and unprecedent focus on the topic that should be useful to researchers interested in scientific developments of ionotropic-gelled chitosan flat structures.

## Figures and Tables

**Table 1:** Polymers and polyelectrolytes used in ionotropic gelation. Adapted from [160] under open access CC-BY license.

Natural Polymers	Synthetic Monomers/Polymers	Multivalent Cations/Anions
Chitosan	Hydroxyethyl methacrylate	Ca^2+^, Mo_7_O_2_^6−^, (PW_12_O_40_)^3−^
Alginate	*N*-(2-Hydroxypropyl) methacrylate	K^+^
Fibrin	*N*-Vinyl-2-pyrrolidone	Fe^2+^, Ba^2+^, Na^+^, Mg^2+^
Collagen	*N*-isopropylacrylamide	Al^3+^
Gelatin	Vinyl acetate	Zn^2+^
